# Variance analysis as a method to predict the locus of plasticity at populations of non-uniform synapses

**DOI:** 10.3389/fncel.2023.1232541

**Published:** 2023-07-17

**Authors:** Lucas B. Lumeij, Aile N. van Huijstee, Natalie L. M. Cappaert, Helmut W. Kessels

**Affiliations:** Cellular and Computational Neuroscience, Swammerdam Institute for Life Sciences, Amsterdam Neuroscience, University of Amsterdam, Amsterdam, Netherlands

**Keywords:** synapse, hippocampus, variance, uniformity, amyloid–beta, excitatory postsynaptic current (EPSC)

## Abstract

Our knowledge on synaptic transmission in the central nervous system has often been obtained by evoking synaptic responses to populations of synapses. Analysis of the variance in synaptic responses can be applied as a method to predict whether a change in synaptic responses is a consequence of altered presynaptic neurotransmitter release or postsynaptic receptors. However, variance analysis is based on binomial statistics, which assumes that synapses are uniform. In reality, synapses are far from uniform, which questions the reliability of variance analysis when applying this method to populations of synapses. To address this, we used an *in silico* model for evoked synaptic responses and compared variance analysis outcomes between populations of uniform versus non-uniform synapses. This simulation revealed that variance analysis produces similar results irrespectively of the grade of uniformity of synapses. We put this variance analysis to the test with an electrophysiology experiment using a model system for which the loci of plasticity are well established: the effect of amyloid-β on synapses. Variance analysis correctly predicted that postsynaptically produced amyloid-β triggered predominantly a loss of synapses and a minor reduction of postsynaptic currents in remaining synapses with little effect on presynaptic release probability. We propose that variance analysis can be reliably used to predict the locus of synaptic changes for populations of non-uniform synapses.

## Introduction

Synaptic plasticity is a crucial mechanism for the brain to adapt behavior based on experience (Kessels and Malinow, 2009). Specifically, strengthening and weakening of hippocampal synapses play a pivotal role in memory formation and forgetting (Martin et al., 2000). However, when a change in synaptic strength occurs, it is often unknown what the underlying mechanism and locus of that change is, e.g., whether this change is either presynaptic or postsynaptic.

The efficacy of synaptic communication is determined by three main parameters: the number of functional vesicle release sites (N), the probability of presynaptic vesicle release per release site (P_*r*_) and the postsynaptic response size to the release of a single vesicle of neurotransmitter (i.e., a quantum), which is called the quantal size (Q) (Korn and Faber, 1991). In the central nervous system, neurotransmitter is released stochastically, leading to fluctuations in postsynaptic responses that roughly follow a binomial distribution when axon bundles are repeatedly stimulated (Korn and Faber, 1991). Quantal analysis on the variance in synaptic responses can be used to predict changes in N, P_*r*_, and Q (Del Castillo and Katz, 1954). If one assumes a binomial distribution, the mean amplitude of postsynaptic responses (μ) and its variance (σ^2^) can be calculated using the following equations:


(1) $$\mu =N\,{\text{P}}_{r}\,Q$$

(2) $$\sigma^{2}=N\,{\text{P}}_{r}\,\left(1-{\text{P}}_{r}\right)\,Q^{2}$$

To dissect which of these three parameters is affected when synaptic changes occur, simple indices can be derived from these equations. Firstly, the inverse square of the coefficient of variation (1/CV^2^) is revealing, because it is independent of Q (Bekkers and Stevens, 1990; Malinow and Tsien, 1990; Redman, 1990):


(3) $$\frac{1}{C\,V^{2}}=\frac{\mu^{2}}{\sigma^{2}}=\frac{N\,{\text{P}}_{r}}{1-{\text{P}}_{r}}$$

The 1/CV^2^ has been used extensively to predict whether a synaptic change was presynaptic (P_*r*_) or postsynaptic (Q) in origin, provided that the number of functional release sites (N) stays constant within an experiment (Bekkers and Stevens, 1990; Malinow and Tsien, 1990). The variance-to-mean ratio (VMR) is a useful index to further dissect the synaptic loci, because it is independent of N (Lupica et al., 1992; van Huijstee and Kessels, 2020):


(4) $$V\,M\,R=\frac{\sigma^{2}}{\mu }=\left(1-{\text{P}}_{r}\right)\,Q$$

Applying the combination of 1/CV^2^ and VMR on evoked synaptic responses has been validated as a method to decipher the contributions of N, P_*r*_, and Q to a change in synaptic strength (van Huijstee and Kessels, 2020; Hogrefe et al., 2022).

There are a number of assumptions underlying a binomial release model (Brock et al., 2020), of which the first two are largely met at central synapses. Firstly, variance analysis assumes that at most one quantum is released at each functional release site per action potential. Most central synapses indeed release at most one vesicle per action potential, although multivesicular release within a single synapse, and even within a single active zone, can occur at central synapses (Korn and Faber, 1991; Quastel, 1997; Oertner et al., 2002; Conti and Lisman, 2003; Jensen et al., 2019; Maschi and Klyachko, 2020; Dürst et al., 2022). Therefore, in situations where multivesicular release is prevalent, one should regard N as the number of functional release sites rather than the number of synapses. A second assumption is that the release of a vesicle happens independently from other release sites, meaning that released quanta summate linearly. This assumption appears to be largely true, since release sites are considered to act autonomously (Ventura and Harris, 1999; Oertner et al., 2002; Christie and Jahr, 2006; Dürst et al., 2022).

However, the assumption related to variance analysis that is clearly not met is that P_*r*_ and Q are uniform across synapses. In fact, previous studies reported a large variety in P_*r*_ between release sites (Hessler et al., 1993; Rosenmund et al., 1993; Dobrunz and Stevens, 1997; Oertner et al., 2002; Dürst et al., 2022). Similarly, the postsynaptic response (Q) to each released vesicle varies between release sites, as Q depends on receptor density and receptor conductance (Dobrunz and Stevens, 1997; Hanse and Gustafsson, 2001), although the amount of neurotransmitter released per vesicle is thought to be relatively uniform (Rost et al., 2015; Dürst et al., 2022). Based on these considerations, we questioned whether this non-uniform distribution of P_*r*_ and Q would cause extra variance to the synaptic responses, making variance analysis potentially unreliable for predicting changes at populations of synapses.

In this study we aimed to test the effects of a non-uniform distribution of P_*r*_ and Q in populations of synapses on the outcome parameters of variance analysis (i.e., 1/CV^2^ and VMR). We did this by simulating whole-cell patch clamp experiments, to study the variance in evoked excitatory postsynaptic currents (EPSCs) in a controlled manner. Comparing uniform and non-uniform input parameters N, P_*r*_, and Q *in silico* and testing their effects on μ, 1/CV^2^, and VMR, allows us to assess the importance of the assumption that synaptic populations should be uniform when conducting variance analysis. To validate our model, we compared the outcomes of our *in silico* model with an actual patch clamp experiment on AMPA receptor (AMPAR) currents in hippocampal CA1 pyramidal neurons receiving Schaffer collateral input from CA3 neurons (i.e., Sc-CA1 synapses) (Kessels et al., 2013). In these experiments, we studied the effect of the expression of the amyloid precursor protein (APP) on the AMPAR EPSCs in CA1 neurons in organotypic hippocampal rat slices. Dual recordings from pairs of APP-expressing and control neurons were used to assess the effects of the overproduction of amyloid-β (Aβ), an important protein in the pathogenesis of Alzheimer’s disease, on synaptic transmission (Selkoe, 2002). Multiple studies that used this model system show that the production of Aβ oligomers reduces synaptic transmission onto CA1 neurons (Kamenetz et al., 2003; Hsieh et al., 2006; Kessels et al., 2010, 2013; Knafo et al., 2016; Reinders et al., 2016). We tested whether variance analysis can be used to make a prediction about the contributions of changes in N, P_*r*_, and/or Q that cause this decrease in EPSC amplitude. Together, this study provides more insight into the strengths and limitations of variance analysis and shows its merit when predicting pre- and postsynaptic plasticity in the hippocampus and possibly in central synapses overall.

## Materials and methods

### Electrophysiology

Organotypic hippocampal slices were prepared, as previously described, from P6-7 female and male Sprague Dawley rats and kept in culture for 6–13 days (Stoppini et al., 1991; Kessels et al., 2013). APP_*CT100*_ was sparsely expressed using Sindbis viral vectors that were injected into CA1 20–30 h before recording. Sparse expression is relevant to avoid immune responses to viral particles (Uyaniker et al., 2019) and to ensure that the majority of synapses from control neurons are sufficiently separated from Aβ-producing neurons (Wei et al., 2010). On the day of recording a cut was made between CA3 and CA1 to prevent stimulus-induced bursting. Whole-cell recordings were obtained simultaneously from neighboring uninfected and infected CA1 neurons; infected neurons were identified by fluorescence using co-expression of GFP. Two stimulating electrodes were placed 100 μm apart laterally and 200 μm in opposite directions (e.g., 100 and 300 μm) along the apical dendrite in the stratum radiatum (Figure 5A). For the recordings 3- to 5-MΩ pipettes were used containing internal solution of 115 mM cesium methanesulfonate, 20 mM CsCl, 10 mM Hepes, 2.5 mM MgCl_2_, 4 mM Na_2_ATP, 0.4 mM Na_3_GTP, 10 mM sodium phosphocreatine (Sigma), and 0.6 mM EGTA (Amresco), at pH 7.25. During recording, slices were perfused with artificial cerebrospinal fluid containing 119 mM NaCl, 2.5 mM KCl, 26 mM NaHCO_3_, 1mM NaH_2_PO_4_, and 11 mM glucose (pH 7.4), and gassed with 5% CO2/95% O_2_ at 27 °C with 4 mM MgCl_2_, 4 mM CaCl_2_, 4 μM 2-chloroadenosine (Sigma), and 100 μM picrotoxin (Sigma). During each recording, neurons received input from the two stimulating electrodes, sweeps from each individual electrode were 3 s apart. The resulting EPSCs were averaged and count as *n* = 1. AMPAR EPSCs were measured as peak inward currents at −60 mV.

### *In silico* simulation

Evoked whole-cell patch clamp experiments were simulated using MATLAB 2021a. To match the electrophysiological data, experimental groups always consisted of 27 *in silico* neurons. In these simulations, populations of neurons were given values for N, P_*r*_, and Q. The number of synapses (N) differed between 5 and 25, depending on the experiment. Each synapse was assigned a release probability (P_*r*_) between 0 and 1. In a uniform population all synapses had the same P_*r*_, but in a non-uniform population, synapses were randomly assigned a value drawn from a beta distribution with a specific mean P_*r*_ and corresponding standard deviation (SD). An example of randomly drawn P_*r*_ values within such a distribution is depicted in Supplementary Figure 1 (i.e., 27 neurons with 15 synapses each). Regarding quantal response size (Q), each synapse was given a value between 5 and 25 pA, depending on the experiment. Again, in uniform populations Q was the same for each synapse, but in non-uniform populations Q was attributed randomly per synapse from the distribution in the Pearson system with mean, standard deviation, skewness (between 0.75 and 1) and kurtosis (4). An exemplar distribution of randomly drawn Q values for one experiment is depicted in Supplementary Figure 1 (i.e., 27 neurons with 15 synapses each).

Each simulated experiment consisted of 48 sweeps, based on the average number of sweeps used in the electrophysiological experiments (Figure 5A). Each sweep meant the stimulation of one population of synapses that was activated. However, to mimic the stochastic process of neurotransmitter release, a go/no go value was randomly drawn from a uniform distribution in the interval (0,1) for each synapse for each sweep. If this go/no go value was equal to or lower than the P_*r*_ of that synapse a “vesicle was released” and the EPSC amplitude of that synapse would be equal to its Q. If the go/no go value would lead to no release, the EPSC amplitude of that synapse would be 0. Per sweep, the currents of all the synapses in which release took place were summated giving the total EPSC amplitude of that sweep. Per neuron/recording this was done 48 times (i.e., nr. of sweeps), leading to a mean EPSC amplitude and its variance per neuron.

### Variance analysis

For the electrophysiological recordings, variance analysis was performed on the mean EPSC amplitudes and variance of responses to 30–80 sweeps, on average 48 sweeps, per neuron. For the *in silico* neurons, 48 sweeps were used. The EPSC amplitudes per sweep and variance per neuron were used to calculate their mean EPSC amplitude, 1/CV^2^ and VMR (equations 2 and 3). These three values were averaged over 27 neurons per group and compared between conditions. Note that multiplying 1/CV^2^ with VMR per neuron leads to its μ value. As a consequence, 1/CV^2^ and VMR are negatively correlated for both simulated and recorded neurons (Supplementary Figure 2).

### Statistics

Multiple *t*-tests with a Holm-Šídák correction were performed on log_2_-normalized data to test whether they differed significantly from 0 or if there were differences between groups. One-way ANOVAs were used to test if there were differences between multiple groups. Paired *t*-tests were used to detect differences between two groups in the electrophysiological experiments and unpaired *t*-tests were used for two-group comparisons in the *in silico* experiments. For all experiments, *p* < 0.05 was considered significant.

## Results

### Variance analysis outcomes are similar between uniform and non-uniform populations of *in silico* synapses

To examine whether a uniform versus a non-uniform input variable distribution (N, P_*r*_ and Q) would lead to different outcomes in variance analysis output (i.e., μ, 1/CV^2^ and VMR), we simulated whole-cell electrophysiological experiments in which excitatory postsynaptic currents are determined by stimulating a population of 27 neurons. In this simulation, for each *in silico* neuron 15 synapses were stimulated (*N* = 15). Release was set to be univesicular, indicating that in this model N represents the number of release sites and also the number of synapses. These *in silico* synapses had physiological values for the mean and standard deviation of the release probability (*P*_*r*_ = 0.3 ± 0.15) and quantal size (*Q* = 15 ± 4.5 pA) based on previous literature for Sc-CA1 hippocampal synapses that receive Schaffer collateral input (Hessler et al., 1993; Rosenmund et al., 1993; Dobrunz and Stevens, 1997; Hanse and Gustafsson, 2001; Oertner et al., 2002). In the non-uniform distributions, P_*r*_ ranged from 0.04 to 0.81 and Q values ranged from and 6 to 28 pA (Supplementary Figure 1). We used these values to design four populations with the same N, mean P_*r*_ and mean Q, but with different distributions for P_*r*_ and Q.

**FIGURE 1:**
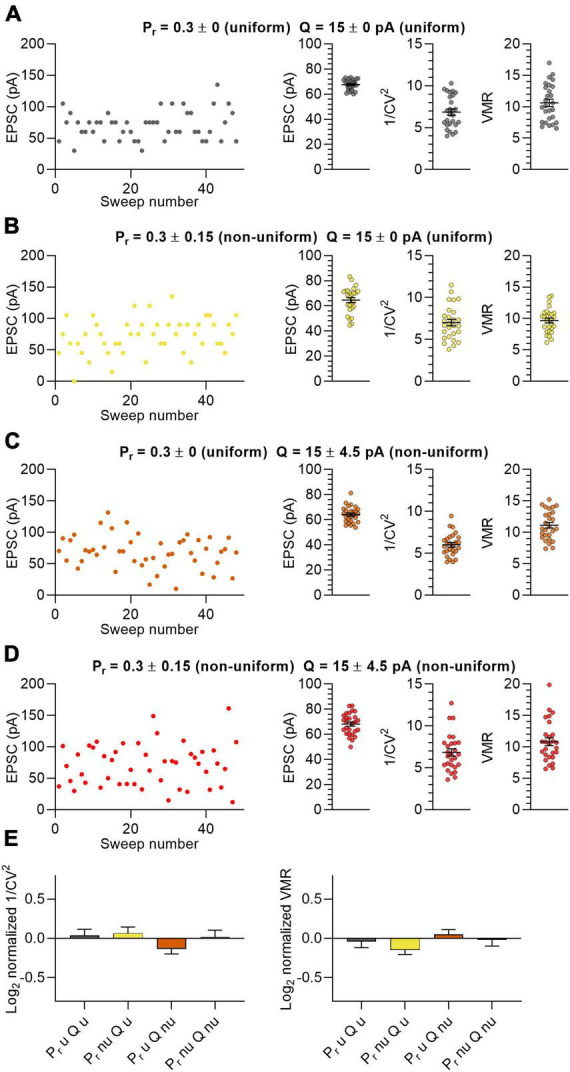
Non-uniform *in silico* populations of synapses do not affect variance analysis outcomes. **(A–D)** Left panel: example traces of summated EPSC amplitudes of 15 stimulated synapses per sweep of one *in silico* neuron. Right panels: average EPSC, 1/CV^2^ and VMR (*n* = 27 neurons). **(A)** Both P_r_ (0.3 ± 0) and Q (15 ± 0 pA) uniform across synapses (grey). **(B)** P_r_ (0.3 ± 0.15) non-uniform and Q uniform (15 ± 0 pA) across synapses (yellow). **(C)** P_r_ (0.3 ± 0) uniform and Q (15 ± 4.5 pA) non-uniform across synapses (orange). **(D)** Both P_r_ (0.3 ± 0.15) and Q (15 ± 4.5 pA) non-uniform across synapses (red). **(E)** Log_2_ values of 1/CV^2^ and VMR for all four conditions normalized to their expected values. Statistics: normalized values were compared to 0 using multiple *t*-tests with a Holm-Šídák correction and to each other by one-way ANOVAs. Error bars indicate SEM.

In the first population, all 15 synapses were uniform having an identical P_*r*_ and Q (Figure 1A; *P*_*r*_ = 0.3 ± 0; *Q* = 15 ± 0 pA). In the second set of *in silico* experiments, we tested groups of 15 synapses per neuron that varied in P_*r*_ but with Q uniform (Figure 1B; Pr = 0.3 ± 0.15; *Q* = 15 ± 0 pA). The third experiment used 15 synapses with uniform P_*r*_ and with different values for Q (Figure 1C; *P*_*r*_ = 0.3 ± 0; *Q* = 15 ± 4.5 pA). In the fourth experiment both P_*r*_ and Q were non-uniform in the 15 synapses (Figure 1D; *P*_*r*_ = 0.3 ± 0.15; *Q* = 15 ± 4.5 pA). Because the N of each neuron and the P_*r*_ and Q of the synapses were on average the same in all four experiments, the average EPSC amplitudes were also highly similar to each other (Figures 1A–D). We calculated the 1/CV^2^ and VMR values for the 4 populations to assess whether the uniformity of the input variables affect the output (Figures 1A–D). We make a distinction between “predicted values” (i.e., 1/CV^2^ and VMR) that result from variance analysis, and “expected values” that are calculated by input variables N, P_*r*_ and Q in equations 1, 3 and 4. Normalizing the predicted values of 1/CV^2^ and VMR to their expected values showed that the prediction did not deviate significantly from the expected outcome for both the uniform and non-uniform populations of *in silico* synapses (Figure 1E). More importantly, these normalized values gave similar values when comparing between the four *in silico* experiments (Figure 1E). Furthermore, changing the distribution further by using either smaller or larger values as standard deviation for P_*r*_ and Q also did not affect the average EPSC amplitude, 1/CV^2^ or VMR significantly (Supplementary Figure 3). These data indicate that the uniformity of a population of synapses did not affect the outcomes of variance analysis.

### Variance analysis correctly predicts changes in N, p_*r*_, and Q in both uniform and non-uniform populations of *in silico* synapses

**FIGURE 2:**
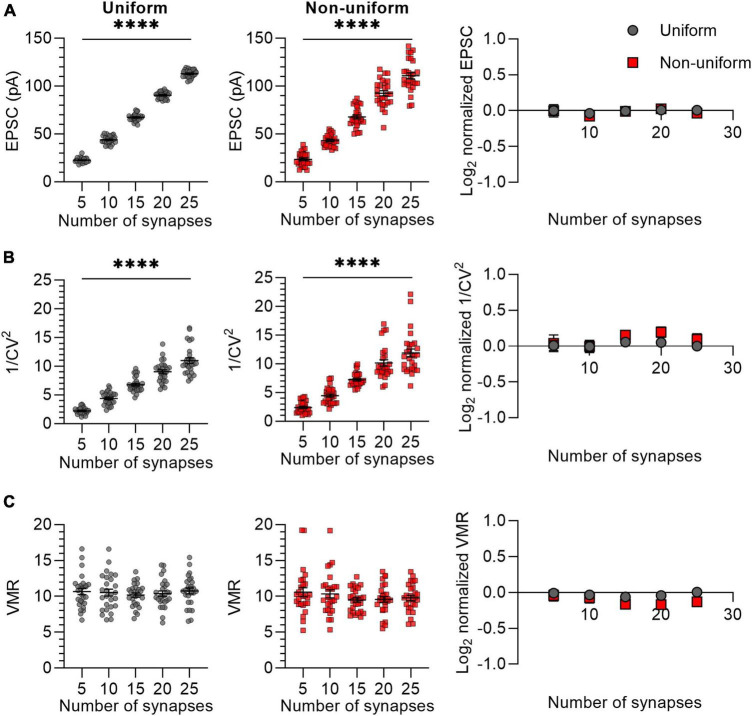
Changes in N lead to similar variance analysis outcomes in both uniform and non-uniform *in silico* populations of synapses. **(A–C)** Left panels: effects of changes in number of synapses (N) on average EPSC **(A)**, 1/CV^2^ **(B)**, and VMR **(C)** in uniform (grey circles) and non-uniform (red squares) populations. Right panel: average log_2_ values of EPSC **(A)**, 1/CV^2^ **(B)**, and VMR **(C)** normalized to expected values of uniform (grey circles) and non-uniform populations (red squares). For uniform populations P_r_ = 0.3 ± 0 and *Q* = 15 ± 0 pA; for non-uniform populations *P*_r_ = 0.3 ± 0.15 and *Q* = 15 ± 4.5 pA (*n* = 27). Statistics: effect of number of synapses on average EPSC, 1/CV^2^ and VMR values (left panels) were tested using one-way ANOVAs. Normalized values were compared to 0 and between uniform and non-uniform using multiple *t*-tests with a Holm-Šídák correction. Error bars indicate SEM; ^****^p < 0.0001.

In the previous experiment, we simulated synaptic responses upon stimulation of 15 Sc-CA1 synapses per neuron. It may be possible that when stimulating a lower number of synapses, differences in variance between uniform and non-uniform populations of synapses become more apparent. To examine the effect of changing the number of synapses, we chose different numbers for N ranging from 5 to 25 with P_*r*_ (0.3) and Q (15 pA) kept constant (Figure 2). We compared synapses that were uniform in both P_*r*_ (0.3 ± 0) and Q (15 ± 0 pA) or non-uniform in both P_*r*_ (0.3 ± 0.15) and Q (15 ± 4.5 pA). The mean EPSC and 1/CV^2^ changed linearly with a change in N (Figures 2A, B), whereas the VMR was not affected by changes in N (Figure 2C), indicating that changing N has the expected effects on the variance analysis parameters in both uniform and non-uniform populations of synapses. The values for average EPSC amplitude, 1/CV^2^ and VMR normalized to their expected values showed that the uniform and the non-uniform populations did not deviate from each other (Figures 2A–C), indicating the number of stimulated synapses does not influence the reliability of variance analysis.

**FIGURE 3:**
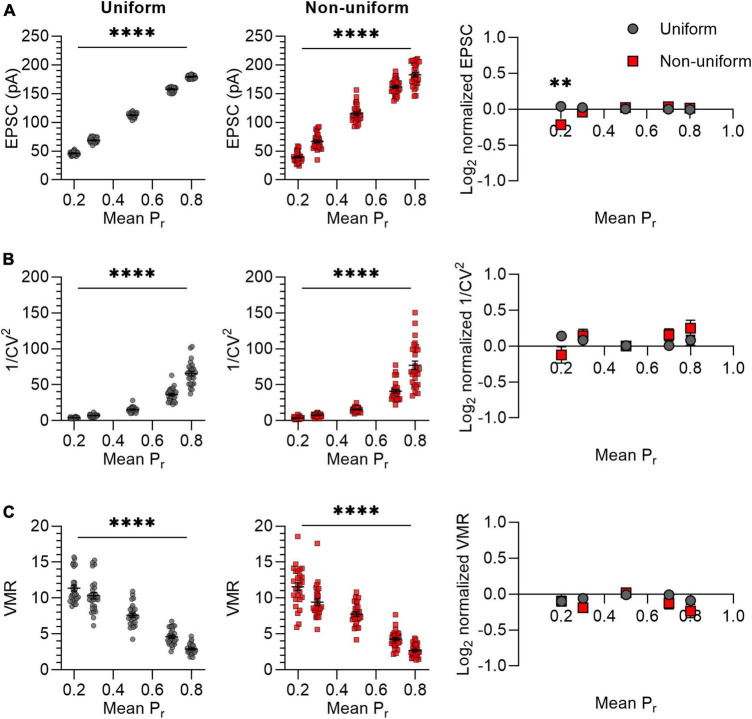
Changes in P_r_ lead to similar variance analysis outcomes in both uniform and non-uniform *in silico* populations of synapses. **(A–C)** Left panels: effects of changes in release probability (P_r_) on average EPSC **(A)**, 1/CV^2^ **(B)**, and VMR **(C)** in uniform (grey circles) and non-uniform (red squares) populations. Right panel: average log_2_ values of EPSC **(A)**, 1/CV^2^ **(B)**, and VMR **(C)** normalized to expected values of uniform (grey circles) and non-uniform populations (red squares). For all populations *N* = 15 and *Q* = 15 pA; in uniform populations SDs of P_r_ and Q were 0; in non-uniform populations SDs of P_r_ were 0.15 (0.14 for *P*_r_ = 0.2 and 0.8) and SDs of Q were 4.5 pA (*n* = 27). Statistics: effect of release probability on average EPSC, 1/CV^2^ and VMR values (left panels) were tested using one-way ANOVAs. Normalized values were compared to 0 and between uniform and non-uniform using multiple *t*-tests with a Holm-Šídák correction. Error bars indicate SEM; ^**^p < 0.01, ^****^p < 0.0001.

It was previously suggested that variance analysis outcomes for non-uniform populations of synapses would deviate more from expected values at high release probability than at low release probabilities (Silver et al., 1998). We therefore asked whether variance analysis comparisons between uniform and non-uniform populations of synapses depend on average release probability. The effects of changes in release probability were assessed by selecting five values for P_*r*_ ranging from 0.2 to 0.8 (Figure 3). Note that we avoided including *in silico* synapses with a P_*r*_ lower than 0 or higher than 1 by setting the standard deviation for non-uniform populations to 0.14 instead of 0.15 for *P*_*r*_ = 0.2 and 0.8. In accordance with the prediction, the mean EPSC increased proportionally with an increase in P_*r*_ for both uniform and non-uniform groups of synapses (Figure 3A). However, only for a low average release probability (*P*_*r*_ = 0.2), the average EPSC amplitude was significantly lower for non-uniform synapses compared with uniform synapses (*p* = 0.001; Figure 3A). This result was unexpected, since the average EPSC should be similar when average N, P_*r*_ and Q are the same. Repeating this *in silico* experiment did give similar average EPSC amplitudes for changes at P_*r*_ = 0.2 (*p* = 0.924; Supplementary Figure 4) and for all other outcome values, which supports the notion that statistical differences can be based on chance. Nevertheless, irrespectively of having obtained a significant difference in EPSC amplitude at average P_*r*_ = 0.2, 1/CV^2^ increased exponentially with an increase in P_*r*_ without differences between the uniform and non-uniform populations of synapses (Figure 3B). In addition, increases in P_*r*_ lead to the expected linear decrease in VMR and also here no differences were found between uniform and non-uniform populations (Figure 3C). This simulation indicates that changes in average P_*r*_ resulted in expected changes in 1/CV^2^ and VMR in both uniform and in non-uniform populations of *in silico* synapses.

**FIGURE 4:**
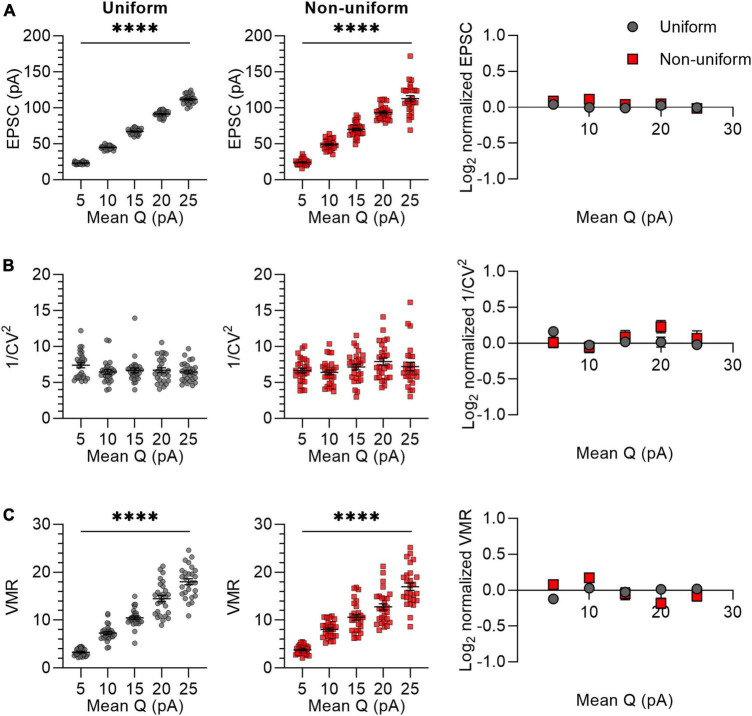
Changes in Q lead to similar variance analysis outcomes in both uniform and non-uniform *in silico* populations of synapses. **(A–C)** Left panels: effects of changes in quantal size (Q) on average EPSC **(A)**, 1/CV^2^ **(B)**, and VMR **(C)** in uniform (grey circles) and non-uniform (red squares) populations. Right panel: average log_2_ values of EPSC **(A)**, 1/CV^2^ **(B)**, and VMR **(C)** normalized to expected values of uniform (grey circles) and non-uniform (red squares) populations. For all populations *N* = 15 and *P*_r_ = 0.3; in uniform populations SDs of Pr and Q were 0; in non-uniform populations SDs of P_r_ were 0.15 and SDs of Q were 4.5 pA (2.5 pA for *Q* = 5 pA) (*n* = 27). Statistics: effect of quantal size on average EPSC, 1/CV^2^ and VMR values (left panels) were tested using one-way ANOVAs. Normalized values were compared to 0 and between uniform and non-uniform using multiple *t*-tests with a Holm-Šídák correction. Error bars indicate SEM; ^****^p < 0.0001.

We next selectively varied the quantal response size by varying the Q from 5 to 25 pA in steps of 5 pA, with a standard deviation of 0 (uniform) or 4.5 (non-uniform) (Figure 4). We chose these values of average Q (15 pA) and standard deviation (4.5 pA) based on previous literature (Dobrunz and Stevens, 1997; Hanse and Gustafsson, 2001). To prevent the inclusion of synapses with negative values for Q, in the non-uniform populations the SD for the lowest value (*Q* = 5 pA) was set to 2.5 instead of 4.5. The average EPSC of synaptic responses increased proportionally with an increase in Q and did not differ between uniform and non-uniform populations for any of the Q values (Figure 4A). The 1/CV^2^ is expected to be independent of Q, which was indeed reflected by variance analysis of both uniform and non-uniform populations of synapses (Figure 4B). VMR values increased linearly with Q and similarly for uniform and non-uniform populations of *in silico* synapses (Figure 4C), which is in line with the expectation that changes in Q are reflected in altered VMR values.

**FIGURE 5:**
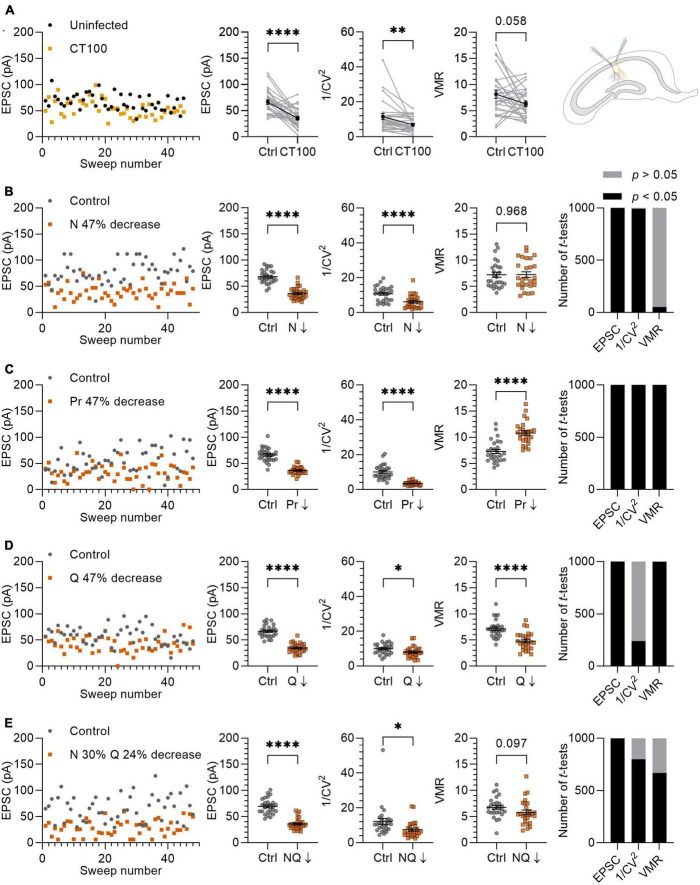
Variance analysis predicts the effects of Aβ on synapses. **(A)** Left panel: example traces of EPSCs from uninfected neuron (black circles) and infected neighboring neuron expressing APP_CT100_ (yellow squares). Middle panels: with averages of EPSC, 1/CV^2^ and VMR in uninfected neurons and their neighboring APP_CT100_-infected neurons. Right panel: schematic representation of two CA1 neurons (uninfected and infected) that are recorded simultaneously. **(B–E)** Left panel: example traces of *in silico* control neuron (grey circles; *N* = 10, *P*_r_ = 0.46 ± 0.23, *Q* = 15 ± 4.5 pA) and test neuron in which N, P_r_, and Q are changed to achieve a ∼47% reduction in EPSC amplitude (orange squares). Middle panels: averages of EPSC, 1/CV^2^ and VMR between control and test neurons (*n* = 27). Right panel: simulations were run 1,000 times and differences in EPSC, 1/CV^2^ and VMR were assessed using *t*-tests and checked for statistical significance. **(B)** N is reduced by ∼47% (*N* = 5); P_r_ and Q are unchanged compared to control. **(C)** P_r_ is reduced by 47% (*P*_r_ = 0.24 ± 0.15); N and Q are unchanged compared to control. **(D)** Q is reduced by 47% (*Q* = 7.95 ± 4.5 pA); N and P_r_ are unchanged compared to control. **(E)** N is decreased by 30% (*N* = 7) and Q is decreased by 24% (*Q* = 11.36 ± 4.5 pA), P_r_ is unchanged compared to the control. Statistics: paired *t*-test **(A)**; unpaired *t*-test **(B–D)**. Error bars indicate SEM; *p < 0.05; ^**^p < 0.01, ^****^p < 0.0001.

Combined, these results indicate that variance analysis correctly predicts changes in N, P_*r*_ and Q for non-uniform groups of *in silico* synapses.

### Validation of variance analysis to predict effects of amyloid-β on synapses

To validate variance analysis as a predictor for the locus of synaptic plasticity, we previously demonstrated in electrophysiological experiments that changing a single parameter, i.e. either N, P_*r*_ or Q individually, resulted in the expected changes in 1/CV^2^ and VMR (van Huijstee and Kessels, 2020). To further investigate the value of variance analysis in a more complex situation where potentially more than one parameter may be altered, we compared our *in silico* model to a previously published *ex vivo* whole-cell patch clamp experiment in which synaptic transmission is affected by the production of Aβ (Kessels et al., 2013). To induce elevated Aβ levels in CA1 pyramidal neurons, organotypic hippocampal slices were injected with viral vectors expressing APP_*CT100*_, the β-secretase product of APP and substrate for Aβ after γ-secretase cleavage. Dual whole-cell recordings from pairs of neighboring uninfected and infected neurons were performed, and AMPAR currents at Sc-CA1 synapses were evoked by stimulating the same axonal input to both neurons. Excitatory transmission was on average 47% lower in APP_*CT100*_-infected CA1 neurons compared with their neighboring control neurons (*p* < 0.0001; Figure 5A). We subsequently tested whether variance analysis could predict a synaptic locus of the observed synaptic depression. We found that the 1/CV^2^ decreased significantly in these recordings by 40% (*p* = 0.0056; Figure 5A). The VMR also tended to decrease by on average 23%, but did not reach statistical significance (*p* = 0.058; Figure 5A).

To further entangle the prediction for a synaptic locus, we reproduced the 47% decrease of the EPSC amplitude *in silico* by lowering N, P_*r*_ and Q separately by ∼47% (Figures 5B–D). In these experiments, we attempted to use values for N, P_*r*_, and Q that would match the electrophysiological experiments in organotypic slices of the rat hippocampus. An important factor here is that the *ex vivo* slice recordings were conducted with 4 mM extracellular Ca^2+^ and P_*r*_ is known to depend strongly on extracellular Ca^2+^ concentration (Dodge and Rahamimoff, 1967; Rosenmund et al., 1993; Dobrunz and Stevens, 1997; Oertner et al., 2002). To estimate P_*r*_ in our *in silico* experiments, we used the VMR of the uninfected neurons (Figure 5A) and assumed that Q was on average 15 pA, since Q is not affected by changing Ca^2+^ concentrations when Mg^2+^ levels are kept high at 4 mM (Hardingham et al., 2006). By using this VMR (8.13) and Q (15 pA) in equation 4, we calculated a P_*r*_ of ∼0.46. This value is approximately in line with the relationship between Ca^2+^ concentration and P_*r*_ reported in literature (Rosenmund et al., 1993; Dobrunz and Stevens, 1997; Oertner et al., 2002). A factor that was considered to influence variance analysis when comparing EPSC recordings with *in silico* results is random electrical noise. When we included noise with a bandwidth of 10 pA to the *in silico* model by adding a random value between +5 pA and −5 pA to the amplitude generated by each sweep, the 1/CV^2^ and VMR values are minimally affected except for recordings with low average EPSC amplitudes (Supplementary Figure 5).

To simulate a loss of functional synapses as the cause of ∼47% decrease in EPSC, we analyzed the effect of lowering N from 10 to 5 synapses per neuron, which resulted in a 43% decrease in the 1/CV^2^ while the VMR remained unchanged (Figure 5B). Decreasing P_*r*_ from 0.46 ± 0.23 to 0.24 ± 0.15 to achieve a 47% decrease in EPSC amplitude led to a 65% decrease in 1/CV^2^ and a 49% increase in VMR (Figure 5C), which particularly for VMR does not match experimental results. When decreasing Q by 47% from 15 to 7.96 pA, we found that 1/CV^2^ decreased by 20% and VMR decreased by 33% (Figure 5D). This result is partially in line with expectation, since the significant change in 1/CV^2^ (*p* = 0.029) unexpectedly predicts a decrease in N or P_*r*_. An advantage of *in silico* experiments over electrophysiology experiments is that they can be effortlessly repeated many times. To assess the probability of finding statistically significant differences, we ran experiments of Figures 5B–D and subsequent statistics for each parameter (EPSC, 1/CV^2^, VMR) 1,000 times. Whereas comparisons were either statistically significant or non-significant in nearly all repetitions for Figures 5B, C, the change in 1/CV^2^ upon a decrease in Q reached significance in only 239 out of 1,000 repetitions (Figure 5D). In conclusion, this variance analysis predicts that APP_*CT100*_ expression predominantly causes a loss of functional release sites, with possibly also a contribution of a decrease in quantal size at remaining functional release sites, and little change in presynaptic release probability.

For a more tailored reproduction, we based our *in silico* parameters on previous findings in similar models to the experiment described here (Kessels et al., 2013). APP and APP_*CT100*_ expression consistently cause a ∼30% spine loss in CA1 dendrites across different studies (Hsieh et al., 2006; Kessels et al., 2010; Reinders et al., 2016). APP expression was reported not to affect presynaptic release probability in Sc-CA1 synapses, shown by an absence of change in paired-pulse facilitation (Kamenetz et al., 2003). There is also evidence that APP expression causes AMPAR removal in remaining Sc-CA1 synapses. Specifically, a ∼25% spine surface reduction of GluA1-expressing AMPARs was found (Hsieh et al., 2006), which predominantly contribute to AMPAR currents (Renner et al., 2017). This observation indicates a decrease in Q in the remaining synapses that did not undergo spine loss. Based on these findings we decided to decrease N by 30% (from 10 to 7 synapses) and cover the remaining EPSC amplitude reduction by lowering Q by 24% (from 15 to 11.36 ± 4.5 pA) (Figure 5E). With these manipulations, 1/CV^2^ decreased significantly by 39% (*p* = 0.019) and VMR decreased by 15%, without reaching statistical significance (*p* = 0.097). Repeating this *in silico* experiment a 1,000 times, 1/CV^2^ lowered significantly in 798/1,000 repetitions and the VMR lowered significantly in 667/1,000 repetitions (Figure 5E). These results obtained by *in silico* simulations approach the biological electrophysiology data (Figure 5A), demonstrating the validity of variance analysis for predicting the locus of synaptic changes.

## Discussion

We show that a non-uniform distribution of release probability and postsynaptic response size in a population of synapses does not affect the outcomes of variance analysis. Testing this assumption is relevant because uniformity is implausible for any population of central synapses (Hessler et al., 1993; Rosenmund et al., 1993; Dobrunz and Stevens, 1997; Hanse and Gustafsson, 2001; Oertner et al., 2002; Dürst et al., 2022). Therefore, non-uniform populations would violate the binomial release model. We intuitively anticipated observing larger variance in synaptic responses for non-uniform populations of synapses in comparison to uniform synapses, thus the model requiring a multinomial instead of a binomial fit. Previous studies used elegant mathematical solutions by incorporating intrasynaptic and intersynaptic quantal variance of P_r_ and Q, thereby incorporating a multinomial model and extending equations for variance analysis (Frerking and Wilson, 1996; Silver et al., 1998). However, our simulation shows that incorporating these factors into the equations is in practice not necessary and that simple indices for 1/CV^2^ and VMR do comply with non-uniform populations of synapses. Therefore, the use of binomial statistics and 1/CV^2^ and VMR to predict the synaptic locus of plasticity is justified in the physiological context of hippocampal synapses. Importantly, our model to test the effects of non-uniformity was validated by testing effects of changes in N, P_r_, and Q on variance analysis output parameters, as these output parameters did not deviate from the expected outcomes in either the uniform or the non-uniform populations. Note that non-uniform populations of *in silico* synapses show a larger range in outcomes for EPSC amplitude compared with uniform populations, without seeing this larger spread for 1/CV^2^ and VMR. This indicates that non-uniformity does lead to a larger variability in results for EPSCs, but that the change in variance relative to EPSC amplitude (i.e., 1/CV^2^ and VMR) remains largely unchanged in non-uniform versus uniform populations.

We argue that variance analysis can be used to predict whether a change in synaptic strength is of pre- or postsynaptic origin, and our *in silico* model may be used to help making such predictions. In our simulation, we programmed synapses to release maximally one vesicle per *in silico* synapse. The majority of CA1 synapses in reality contain multiple vesicle docking sites per synapse, which are potential release sites that operate independently (Schikorski and Stevens, 1997; Rudolph et al., 2015; Pulido and Marty, 2017; Sakamoto et al., 2018). As long as the release probability is sufficiently low that maximally one docked vesicle is released in response to a single action potential, N represents both the number of active release sites as well as the number of active synapses. However, predominantly at large synapses that have many docked vesicles and under conditions that allow high release probabilities, multivesicular release can occur at CA1 synapses (Oertner et al., 2002; Conti and Lisman, 2003; Jensen et al., 2019; Dürst et al., 2022). In addition, in many other types of synapses multivesicular release may be more common than previously thought (Rudolph et al., 2015). For instance, a recent study that used a combination of electron microscopy and variance analysis of electrophysiological recordings demonstrates that in the mouse neocortex the number of release sites appeared to be at least 2.7-fold higher than the number of anatomical synapses (Holler et al., 2021). Therefore, when applying variance analysis on groups of synapses that may have multivesicular release, a decrease in N may not necessarily predict a loss or silencing of synapses (Isaac et al., 1995; Liao et al., 1995; Kerchner and Nicoll, 2008) but instead should be interpreted as presynaptic inactivation of vesicle release sites or postsynaptic silencing of active zones. Another potential factor of caution in interpreting variance analysis data is whether Q solely represents postsynaptic changes or also presynaptic changes. There is evidence that the amount of neurotransmitter stored in vesicles can vary slightly (Hanse and Gustafsson, 2001; Wu et al., 2007; Goh et al., 2011; Takamori, 2016). However, vesicles that are not completely filled have much lower release probabilities (Rost et al., 2015), suggesting that the relationship between the amount of neurotransmitter in a vesicle and its release probability can be a mechanism that ensures quantal uniformity (Rost et al., 2015; Dürst et al., 2022). These studies imply that when variance analysis predicts a change in Q, this can most likely be attributed to postsynaptic plasticity.

As an example to assess the predictive value of variance analysis, we applied it to an experiment that has been used to study the effects of Aβ on synapses. In this model system, CA1 neurons in rat organotypic slices that acutely overproduce APP or APP_CT100_ show reduced synaptic plasticity and a loss of ∼30% of spines at their apical dendrites (Hsieh et al., 2006; Kessels et al., 2010; Wei et al., 2010; Reinders et al., 2016). The remaining synapses in APP_CT100_-expressing CA1 neurons have reduced AMPAR levels, with a substantial loss of GluA3-containing AMPARs and to a lesser extent GluA1-containing ones are removed from synapses (Hsieh et al., 2006). Because GluA3-containing AMPARs contribute little to synaptic currents of CA1 neurons under basal conditions (Renner et al., 2017), the removal of 25% of GluA1-containing AMPARs will predominantly cause a reduced synaptic transmission in these neurons. Although Aβ can affect presynaptic release (Barthet and Mulle, 2020), in this model system release probability is not affected (Kamenetz et al., 2003), likely because APP_CT100_ is only acutely produced at the postsynaptic neuron and not presynaptically. The outcomes of variance analysis in our electrophysiological recordings of APP_CT100_-expressing CA1 neurons are in line with these previous observations. Moreover, if we mimic these effects by reducing N and Q in the *in silico* model, the variance analysis parameters closely match the changes caused by Aβ overproduction in the electrophysiological data. We note that Aβ overproduction appears to mainly target smaller spines, because PSD-95, a prominent synaptic scaffolding protein that is relatively more enriched at large synapses, protects synapses from Aβ (Dore et al., 2021). As a consequence, Aβ overproduction would also change the distribution of P_r_ and/or Q. Yet, as we demonstrate in this study, such a change in distribution does not affect variance analysis results.

We here propose that variance analysis using both 1/CV^2^ and VMR can have a predictive value to assess how a change in synaptic transmission has occurred. Although we here show that variance analysis results are independent of the uniformity of synapses, we remain cautious for using variance analysis to predict absolute values of N, P_r_, or Q. Instead we advocate this method as a useful tool to predict whether a change in synaptic transmission is caused by a change in N, P_r_, and/or Q (van Huijstee and Kessels, 2020). As such, variance analysis can be reliably used as a simple and effective tool to characterize synaptic changes identified in evoked electrophysiological recordings to give direction in further experiments to measure parameters of synaptic plasticity more directly and in a quantitative manner.

## Data availability statement

The raw data supporting the conclusions of this article will be made available by the authors, without undue reservation.

## Ethics statement

The animal study was reviewed and approved by the Animal Experiment Committee, Swammerdam Institute for Life Sciences, University of Amsterdam.

## Author contributions

LL and HK conceptualized the project. LL and AH performed experiments and data analysis. NC wrote the code for the *in silico* model. LL, NC, and HK wrote the manuscript. HK supervised and acquired funding. All authors approved the final manuscript.
