# Supplementary material for: Variance analysis as a method to predict the locus of plasticity at populations of non-uniform synapses
Source: Front Cell Neurosci. 2023 Jul 17;17:1232541. doi: 10.3389/fncel.2023.1232541 (PMC10388551; doi:10.3389/fncel.2023.1232541)
Supplement: Supplementary file 1 [file Data_Sheet_1.PDF]

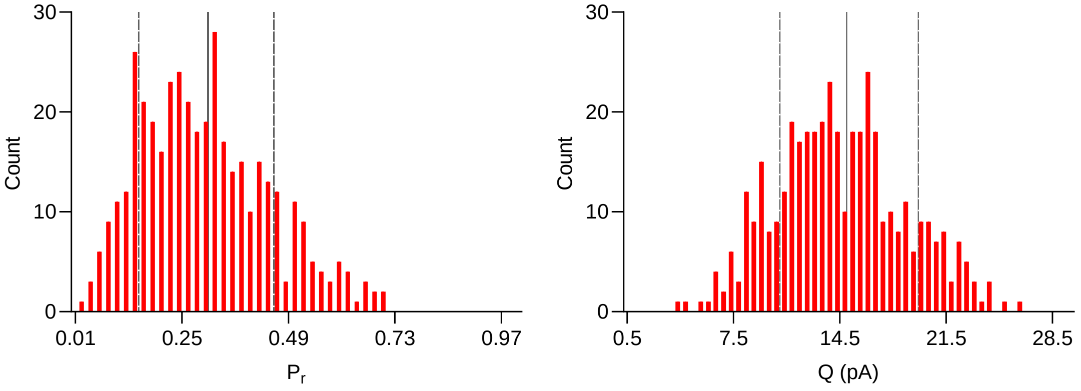

SUPPLEMENTARY FIGURE S1. P_r and Q for non-uniform populations are randomly drawn from physiological distributions. Examples of the randomly drawn values in a beta distribution with $P_r = 0.3 \pm 0.15$ ($\alpha = 2.5$; $\beta = 5.8$) and a distribution in the Pearson system with $Q = 15 \pm 4.5$ pA (skewness = 0.75; kurtosis = 4). In grey mean (solid line) and SDs (dashed lines) are depicted.

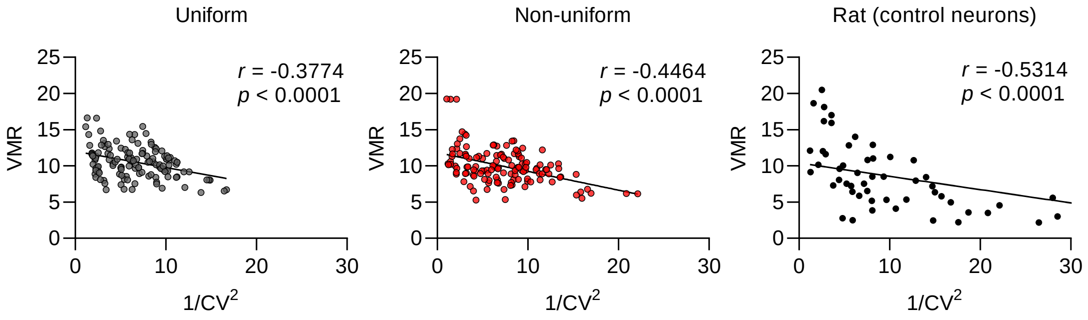

SUPPLEMENTARY FIGURE S2. In both the simulated and the electrophysiological data, $1/CV^2$ and VMR show a negative correlation. Simulated neurons with uniform and non-uniform populations with N ranging from 5 to 25 (Figure 2) were used to compare $1/CV^2$ and VMR values. For the electrophysiological rat data, untreated control neurons were used.

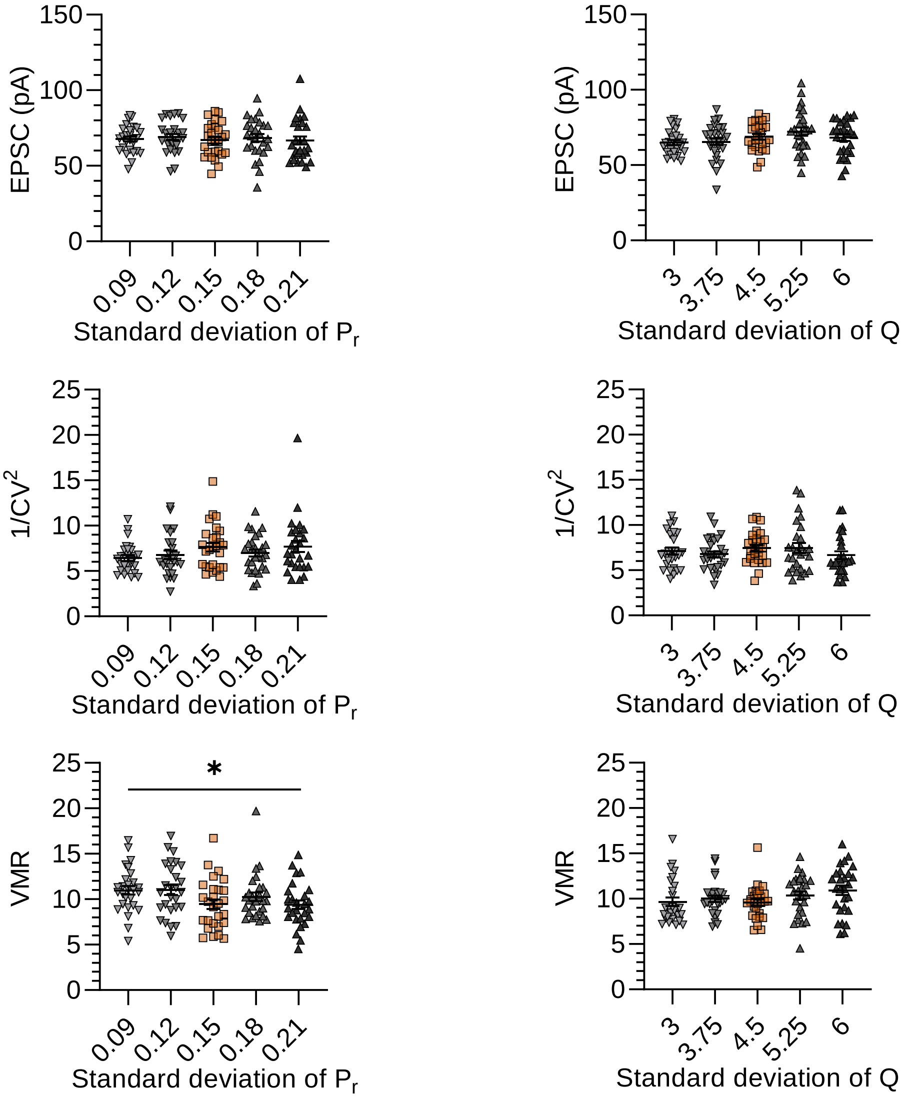

SUPPLEMENTARY FIGURE S3. Changing distributions of P_r and Q does not affect variance analysis outcomes. Effects of different SDs for P_r and Q on EPSC, $1/CV^2$ and VMR. N (15), mean P_r (0.3) and mean Q (15 pA) were kept constant. Statistics: one-way ANOVAs were used to test effect of changing SDs. Error bars indicate SEM; $*p < 0.05$.

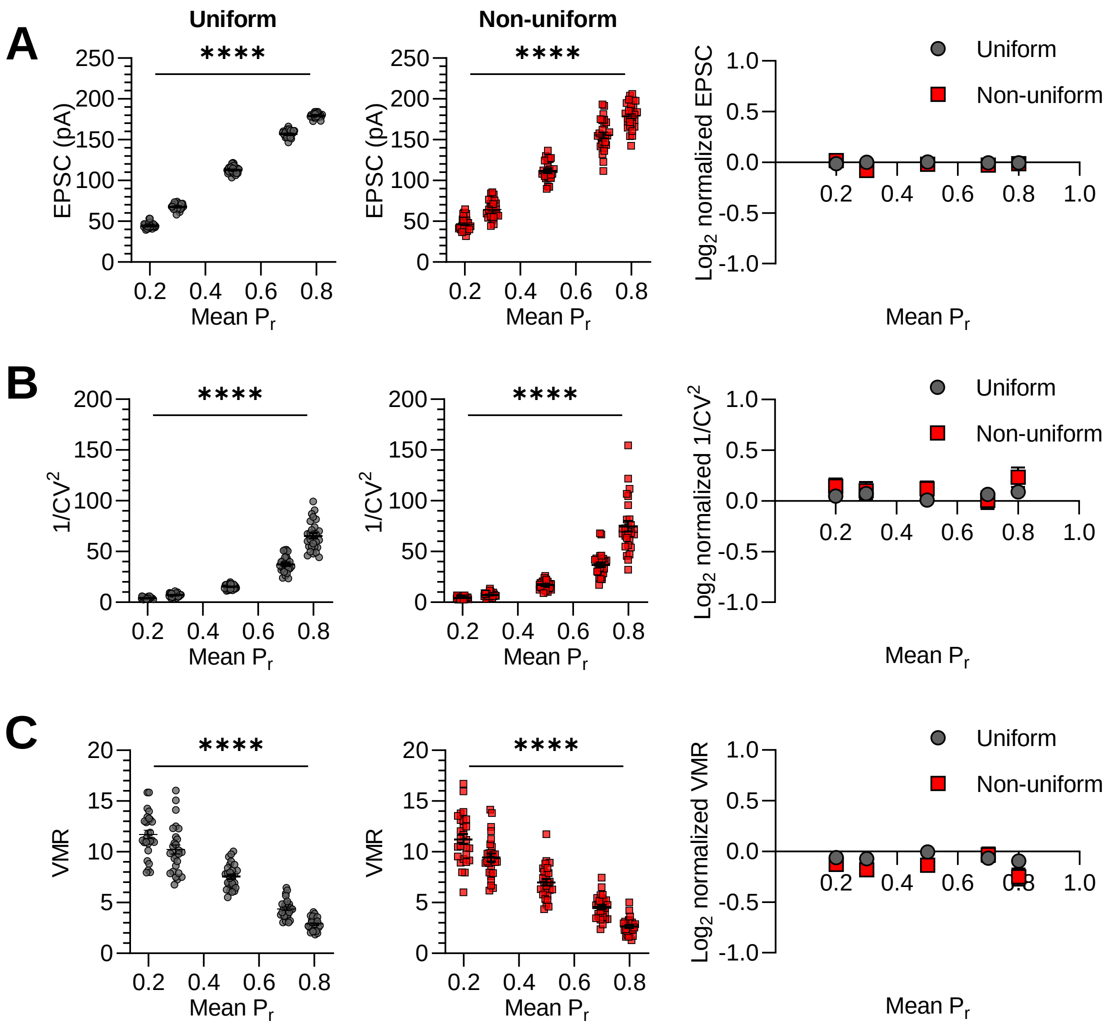

SUPPLEMENTARY FIGURE S4. Repetition of simulating changes in P_r and its effect on variance analysis outcomes (Figure 3). **(A-C)** Left panels: effects of changes in release probability (P_r) on average EPSC **(A)**, $1/CV^2$ **(B)** and VMR **(C)** in uniform (grey circles) and non-uniform (red squares) populations. Right panel: average $\log_2$ values of EPSC **(A)**, $1/CV^2$ **(B)** and VMR **(C)** normalized to expected values of uniform (grey circles) and non-uniform (red squares) populations. For all populations N = 15 and Q = 15 pA; in uniform populations SDs of P_r and Q were 0; in non-uniform populations SDs of P_r were 0.15 (0.14 for $P_r = 0.2$ and 0.8) and SDs of Q were 4.5 pA (n = 27). Statistics: effect of release probability on average EPSC, $1/CV^2$ and VMR values (left panels) were tested using one-way ANOVAs. Normalized values were compared to 0 and between uniform and non-uniform using multiple *t*-tests with a Holm-Šídák correction. Error bars indicate SEM; ****$p < 0.0001$.

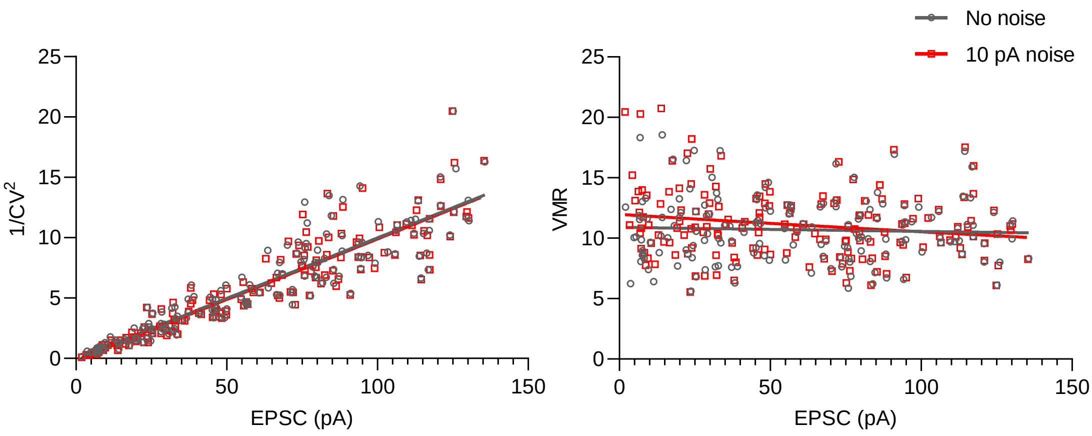

SUPPLEMENTARY FIGURE S5. Intrinsic recording noise affects variance analysis outcomes at low EPSC amplitudes. Neurons with N values ranging from 2 to 25 (in 6 steps), while keeping P_r (0.3) and Q (15 pA) constant, were simulated (*n* = 162; 6*27 neurons). Noise was simulated by adding a random value between -5 and 5 pA to each sweep in each neuron. The EPSC values were compared with $1/CV^2$ and VMR for neurons without noise (grey circles) and the same neurons, but with a random noise value added to their sweep EPSC values (red squares).
